# Crystal structure of *N*-hy­droxy­picolinamide monohydrate

**DOI:** 10.1107/S2056989015024706

**Published:** 2016-01-06

**Authors:** Inna S. Safyanova, Kateryna A. Ohui, Irina V. Omelchenko

**Affiliations:** a Department of Chemistry, National Taras Shevchenko University of Kyiv, Volodymyrska Street 64, 01601 Kiev, Ukraine; b SSI "Institute for Single Crystals", National Academy of Sciences of Ukraine, Lenina ave. 60, Kharkiv 61001, Ukraine

**Keywords:** crystal structure, hydroxamic acids, hydrogen bonds, π–π stacking inter­actions

## Abstract

The crystal structure of the title compound, C_6_H_6_N_2_O_2_·H_2_O, consists of *N*-hy­droxy­picolinamide and water mol­ecules connected through O—H⋯O and N—H⋯N hydrogen bonds. The O—H⋯O inter­actions and π–π stacking inter­actions between the pyridine rings [centroid–centroid distance = 3.427 (1) Å] organize the components into columns extending along the *b* axis and the N—H⋯N hydrogen bonds link these columns into a two-dimensional framework parallel to (100). The *N*-hy­droxy­picolinamide mol­ecule adopts a strongly flattened conformation and only the O—H group H atom deviates significantly from the mol­ecule best plane. The dihedral angle between the hydroxamic group and the pyridine ring is 5.6 (2)°. The conformation about the hydroxamic group C—N bond is *Z* and that about the C—C bond between the pyridine and hydroxamic groups is *E*.

## Abstract 2

In C_6_H_6_N_2_O_2_·H_2_O, the *N*-hy­droxy­picolinamide mol­ecule adopts a strongly flattened conformation. O—H⋯O inter­actions and π–π stacking inter­actions between the pyridine rings organize the crystal components into columns extending along the *b* axis while N—H⋯N hydrogen bonds link these columns into a two-dimensional framework parallel to (100).

## Chemical context   

Hydroxamic acids (HA) are weak organic acids with the general formula *R*—C(=O)—NH—OH. HA can exist as keto and imino­(enol) tautomers with two isomers, *E* and *Z*, for each form, and in the zwitterionic form (see Scheme below). They have found broad application in coordination chemistry due to their diversity and comparatively facile synthesis (Świątek-Kozłowska *et al.*, 2000 ▸; Dobosz *et al.*, 1999 ▸). In addition, they exhibit biological activities related to their enzyme-inhibitory properties (Marmion *et al.*, 2013 ▸). HAs are widely used in coordination and supra­molecular chemistry as scaffolds in the preparation of metallacrowns (Seda *et al.*, 2007 ▸; Jankolovits *et al.*, 2013 ▸; Safyanova *et al.*, 2015 ▸) and as building blocks of coordination polymers (Gumienna-Kontecka *et al.*, 2007 ▸; Golenya *et al.*, 2014 ▸; Pavlishchuk *et al.*, 2010 ▸, 2011 ▸). 
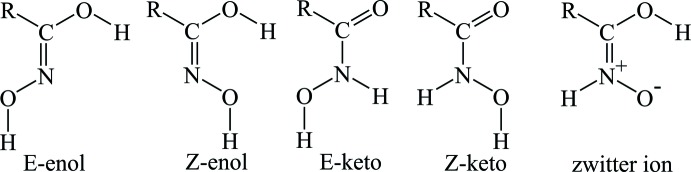


*N*-Hy­droxy­picolinamide, known also as picoline-2-hydroxamic acid (o-PicHA), has been used extensively for the synthesis of polynuclear complexes, especially in the synthesis of diverse metallacrowns (Stemmler *et al.*, 1999 ▸; Seda *et al.*, 2007 ▸; Jankolovits *et al.*, 2013 ▸; Golenya *et al.*, 2012 ▸; Gumienna-Kontecka *et al.*, 2013 ▸). Presently, the Cambridge Structural Database (Groom & Allen, 2014 ▸) contains more than 20 entries of coordination compounds based on *N*-hy­droxy­picolinamide.

Our inter­est in *N*-hy­droxy­picolinamide stems also from the fact that in the course of synthesis of the title and related compounds from 2-picolinic acid esters (Hynes, 1970 ▸), the products are frequently contaminated with impurities that result from hydrolysis of the ester or hydroxamic groups to the carb­oxy­lic group. Structural information about the title compound will be helpful in controlling the purity of the synthesised ligand by powder diffraction.
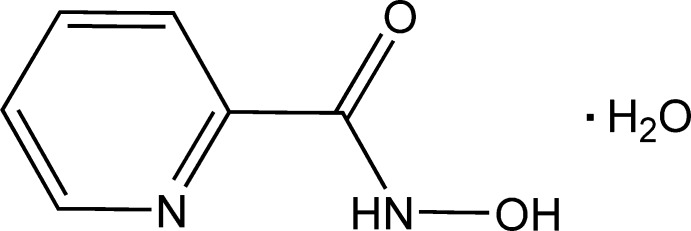


## Structural commentary   

**Figure 1:**
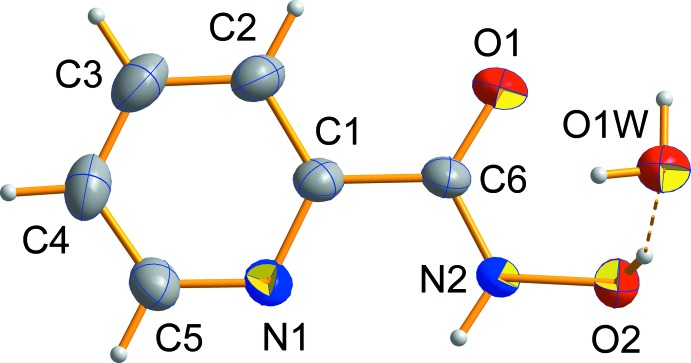
The asymmetric unit of the title compound, with displacement ellipsoids drawn at the 50% probability level. H atoms are shown as spheres of arbitrary radius. The dashed line indicates a hydrogen bond.

The mol­ecular structure of the title compound is presented in Fig. 1 ▸. The crystal structure of the title compound consists of an *N*-hy­droxy­picolinamide mol­ecule in the *Z*-keto tautomeric form in agreement with the C=O and C—N bond lengths [1.234  (2) and 1.325 (2) Å, respectively] and a water mol­ecule. The *N*-hy­droxy­picolinamide mol­ecule adopts a strongly flattened conformation and only the O—H group H atom deviates significantly from the mol­ecular best plane. The maximum deviation from this plane for non-hydrogen atom is 0.083 (1) Å for O1 and the hydroxyl group H2 atom is displaced from the mean plane by 0.80 (1) Å in the direction of the water mol­ecule. The dihedral angle between the hydroxamic group and the pyridine ring is 5.6 (2)°. The configuration about the hydroxamic group C—N bond is *Z* and that about the C—C bond between the pyridine and hydroxamic groups is *E* [torsion angles O2—N2—C6—O1 −0.4 (3)°, N1—C1—C6—O1 175.6 (2)°].

## Supra­molecular features   

**Figure 2:**
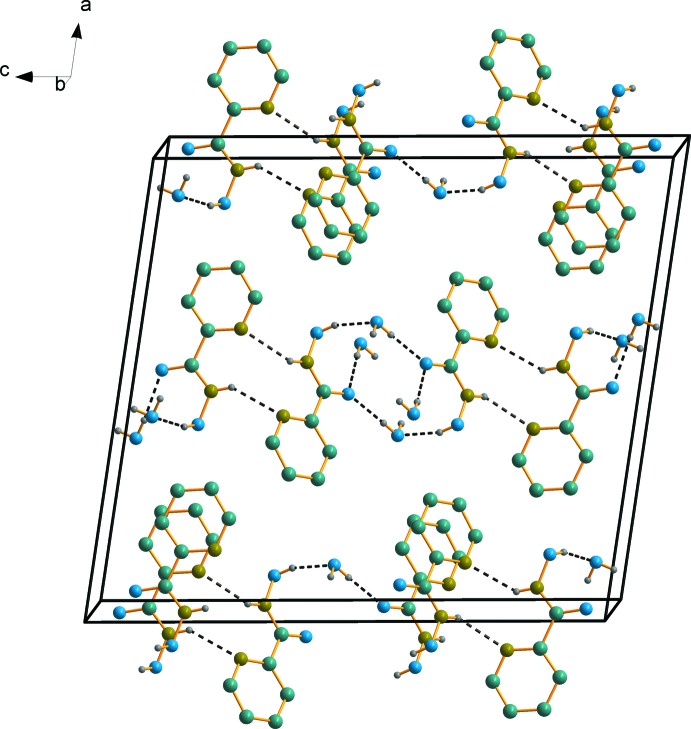
A packing diagram of the title compound. Hydrogen bonds are indicated by dashed lines. H atoms not involved in hydrogen bonding have been omitted for clarity.

The mol­ecular components of the title compound are connected by O—H⋯O and N—H⋯N hydrogen bonds (Table 1 ▸) into a two-dimensional framework parallel to (100) (Fig. 2 ▸). The O—H⋯O inter­actions and π–π stacking inter­actions between the pyridine rings [centroid–centroid distance 3.427 (1) Å] organize the crystal components into columns extending along the *b* axis while the N—H⋯N hydrogen bonds link these columns into a two-dimensional framework parallel to (100) (Fig.2).

## Database survey   

A search of the Cambridge Structural Database (Version 5.36, last update February 2015; Groom & Allen, 2014 ▸) revealed two crystal structures of isomeric pyridine hydroxamic acids and the crystal structure of 2,6-pyridinedi­hydroxamic acid (Golenya *et al.*, 2007 ▸; Makhmudova *et al.*, 2001 ▸; Griffith *et al.*, 2008 ▸).

## Synthesis and crystallization   

The title compound was obtained by the reaction of methyl 2-picolinate and hydroxyl­amine in methanol solution according to a reported procedure (Hynes, 1970 ▸). Colorless crystals suitable for X-ray diffraction were obtained from a methanol solution by slow evaporation at room temperature (yield 79%).

## Refinement   

Crystal data, data collection and structure refinement details are summarized in Table 2 ▸. The crystal was modelled as a non-merohedral twin with the volume ratio of two twin domains refined at 89:19. The C—H, N—H and O—H hydrogen atoms of the organic mol­ecule were found from the difference Fourier maps but for further calculations they were positioned geometrically and constrained to ride on their parent atoms with C—H = 0.93 Å, N—H = 0.86 Å and O—H = 0.82 Å, and with *U*_iso_ = 1.2*U*_eq_(C,N) or *U*_iso_ = 1.5*U*_eq_(O). The H atoms of the water mol­ecule were located in the difference Fourier maps, the O—H distances standardized to 0.85 Å and refined in riding-model approximation with *U*_iso_(H) = 1.5*U*_eq_(O).

## Supplementary Material

Crystal structure: contains datablock(s) I. DOI: 10.1107/S2056989015024706/gk2650sup1.cif

Structure factors: contains datablock(s) I. DOI: 10.1107/S2056989015024706/gk2650Isup2.hkl

Supporting information file. DOI: 10.1107/S2056989015024706/gk2650Isup3.cml

CCDC reference: 1444026

Additional supporting information:  crystallographic information; 3D view; checkCIF report

## Figures and Tables

**Table 1:** Hydrogen-bond geometry (Å, °)

*D*—H⋯*A*	*D*—H	H⋯*A*	*D*⋯*A*	*D*—H⋯*A*
O2—H2⋯O1*W*	0.82	1.86	2.656 (2)	163
N2—H2*A*⋯N1^i^	0.86	2.31	3.010 (2)	139
O1*W*—H1*WA*⋯O1^ii^	0.85	2.14	2.976 (2)	168
O1*W*—H1*WB*⋯O1^iii^	0.85	1.94	2.788 (2)	173

Symmetry codes: (i) 
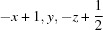
; (ii) 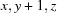; (iii) 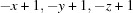.

**Table 2:** Experimental details

Crystal data	
Chemical formula	C_6_H_6_N_2_O_2_·H_2_O
*M*_r_	156.14
Crystal system, space group	Monoclinic, *C*2/*c*
Temperature (K)	298
*a*, *b*, *c* (Å)	18.7471 (13), 3.8129 (4), 20.4813 (17)
β (°)	100.570 (7)
*V* (Å^3^)	1439.2 (2)
*Z*	8
Radiation type	Mo *K*α
μ (mm^−1^)	0.12
Crystal size (mm)	0.4 × 0.4 × 0.1

Data collection	
Diffractometer	Agilent Xcalibur, Sapphire3
Absorption correction	Multi-scan (*CrysAlis PRO*; Agilent, 2013 ▸)
*T*_min_, *T*_max_	0.476, 1.000
No. of measured, independent and observed [*I* > 2σ(*I*)] reflections	2491, 1401, 1053
*R*_int_	0.037
(sin θ/λ)_max_ (Å^−1^)	0.617

Refinement	
*R*[*F* ^2^ > 2σ(*F* ^2^)], *wR*(*F* ^2^), *S*	0.053, 0.143, 0.99
No. of reflections	1401
No. of parameters	102
H-atom treatment	H-atom parameters constrained
Δρ_max_, Δρ_min_ (e Å^−3^)	0.19, −0.25

Computer programs: *CrysAlis PRO* (Agilent, 2013 ▸), *SHELXT* (Sheldrick, 2015*a* ▸), *SHELXL2014* (Sheldrick, 2015*b* ▸) and *OLEX2* (Dolomanov *et al.*, 2009 ▸).

## supplementary crystallographic information

### Crystal data

**Table:**

C_6_H_6_N_2_O_2_·H_2_O	*D*_x_ = 1.441 Mg m^−^^3^
*M**_r_* = 156.14	Melting point: 393 K
Monoclinic, *C*2/*c*	Mo *K*α radiation, λ = 0.71073 Å
*a* = 18.7471 (13) Å	Cell parameters from 893 reflections
*b* = 3.8129 (4) Å	θ = 4.1–29.0°
*c* = 20.4813 (17) Å	µ = 0.12 mm^−^^1^
β = 100.570 (7)°	*T* = 298 K
*V* = 1439.2 (2) Å^3^	Plate, clear colourless
*Z* = 8	0.4 × 0.4 × 0.1 mm
*F*(000) = 656	

### Data collection

**Table:**

Agilent Xcalibur, Sapphire3 diffractometer	1401 independent reflections
Radiation source: Enhance (Mo) X-ray Source	1053 reflections with *I* > 2σ(*I*)
Graphite monochromator	*R*_int_ = 0.037
Detector resolution: 16.1827 pixels mm^-1^	θ_max_ = 26.0°, θ_min_ = 3.3°
ω scans	*h* = −22→22
Absorption correction: multi-scan (*CrysAlis PRO*; Agilent, 2013)	*k* = −4→4
*T*_min_ = 0.476, *T*_max_ = 1.000	*l* = −24→24
2491 measured reflections	

### Refinement

**Table:**

Refinement on *F*^2^	Primary atom site location: structure-invariant direct methods
Least-squares matrix: full	Secondary atom site location: difference Fourier map
*R*[*F*^2^ > 2σ(*F*^2^)] = 0.053	Hydrogen site location: inferred from neighbouring sites
*wR*(*F*^2^) = 0.143	H-atom parameters constrained
*S* = 0.99	*w* = 1/[σ^2^(*F*_o_^2^) + (0.0751*P*)^2^] where *P* = (*F*_o_^2^ + 2*F*_c_^2^)/3
1401 reflections	(Δ/σ)_max_ < 0.001
102 parameters	Δρ_max_ = 0.19 e Å^−^^3^
0 restraints	Δρ_min_ = −0.25 e Å^−^^3^

### Special details

**Table:**

Geometry. All e.s.d.'s (except the e.s.d. in the dihedral angle between two l.s. planes) are estimated using the full covariance matrix. The cell e.s.d.'s are taken into account individually in the estimation of e.s.d.'s in distances, angles and torsion angles; correlations between e.s.d.'s in cell parameters are only used when they are defined by crystal symmetry. An approximate (isotropic) treatment of cell e.s.d.'s is used for estimating e.s.d.'s involving l.s. planes.
Refinement. Refined as a 2-component twin. Refinement of *F*^2^ against ALL reflections. The weighted *R*-factor *wR* and goodness of fit *S* are based on *F*^2^, conventional *R*-factors *R* are based on *F*, with *F* set to zero for negative *F*^2^. The threshold expression of *F*^2^ > σ(*F*^2^) is used only for calculating *R*-factors(gt) *etc*. and is not relevant to the choice of reflections for refinement. *R*-factors based on *F*^2^ are statistically about twice as large as those based on *F*, and *R*- factors based on ALL data will be even larger.

### Fractional atomic coordinates and isotropic or equivalent isotropic displacement parameters (Å^2^)

**Table:**

	*x*	*y*	*z*	*U*_iso_*/*U*_eq_	
O1	0.52649 (8)	0.3700 (4)	0.43254 (7)	0.0468 (5)	
O2	0.40179 (7)	0.5190 (5)	0.35109 (7)	0.0506 (5)	
H2	0.3916	0.6429	0.3808	0.076*	
N1	0.59448 (9)	0.8106 (5)	0.30306 (8)	0.0366 (5)	
N2	0.46909 (9)	0.6163 (5)	0.33806 (8)	0.0410 (5)	
H2A	0.4722	0.7305	0.3025	0.049*	
C1	0.59810 (10)	0.6575 (5)	0.36271 (9)	0.0323 (5)	
C2	0.66231 (11)	0.6152 (6)	0.40743 (11)	0.0428 (6)	
H2B	0.6628	0.5119	0.4487	0.051*	
C3	0.72601 (11)	0.7308 (6)	0.38915 (13)	0.0519 (7)	
H3	0.7703	0.7030	0.4178	0.062*	
C4	0.72296 (11)	0.8863 (6)	0.32857 (13)	0.0482 (6)	
H4	0.7650	0.9656	0.3154	0.058*	
C5	0.65653 (12)	0.9234 (6)	0.28737 (11)	0.0434 (6)	
H5	0.6549	1.0327	0.2465	0.052*	
C6	0.52864 (10)	0.5321 (6)	0.38079 (9)	0.0332 (5)	
O1W	0.39862 (8)	0.9488 (5)	0.45255 (8)	0.0501 (5)	
H1WA	0.4308	1.0938	0.4455	0.075*	
H1WB	0.4202	0.8351	0.4861	0.075*	

### Atomic displacement parameters (Å^2^)

**Table:**

	*U*^11^	*U*^22^	*U*^33^	*U*^12^	*U*^13^	*U*^23^
O1	0.0447 (8)	0.0657 (11)	0.0287 (8)	0.0009 (7)	0.0035 (6)	0.0147 (7)
O2	0.0332 (8)	0.0832 (13)	0.0350 (9)	−0.0121 (8)	0.0051 (6)	0.0062 (8)
N1	0.0370 (9)	0.0465 (11)	0.0267 (9)	−0.0051 (8)	0.0069 (7)	−0.0036 (8)
N2	0.0307 (9)	0.0670 (13)	0.0252 (9)	−0.0038 (8)	0.0048 (7)	0.0107 (9)
C1	0.0344 (10)	0.0371 (11)	0.0252 (10)	0.0012 (8)	0.0047 (8)	−0.0061 (8)
C2	0.0369 (11)	0.0543 (14)	0.0347 (12)	0.0052 (9)	0.0000 (9)	−0.0030 (11)
C3	0.0322 (11)	0.0640 (17)	0.0554 (16)	0.0028 (11)	−0.0027 (10)	−0.0116 (13)
C4	0.0351 (11)	0.0569 (15)	0.0552 (15)	−0.0091 (10)	0.0156 (10)	−0.0148 (12)
C5	0.0435 (12)	0.0538 (15)	0.0348 (12)	−0.0062 (11)	0.0121 (9)	−0.0047 (11)
C6	0.0359 (11)	0.0409 (12)	0.0224 (10)	−0.0012 (9)	0.0043 (8)	−0.0013 (9)
O1W	0.0408 (8)	0.0687 (11)	0.0404 (9)	0.0044 (8)	0.0068 (7)	0.0163 (8)

### Geometric parameters (Å, º)

**Table:**

O1—C6	1.234 (2)	C2—C3	1.387 (3)
O2—N2	1.387 (2)	C2—H2B	0.9300
O2—H2	0.8200	C3—C4	1.367 (3)
N1—C5	1.334 (3)	C3—H3	0.9300
N1—C1	1.344 (3)	C4—C5	1.378 (3)
N2—C6	1.325 (2)	C4—H4	0.9300
N2—H2A	0.8600	C5—H5	0.9300
C1—C2	1.382 (3)	O1W—H1WA	0.8503
C1—C6	1.496 (3)	O1W—H1WB	0.8499
			
N2—O2—H2	109.5	C4—C3—H3	120.4
C5—N1—C1	117.26 (17)	C2—C3—H3	120.4
C6—N2—O2	119.60 (16)	C3—C4—C5	118.9 (2)
C6—N2—H2A	120.2	C3—C4—H4	120.6
O2—N2—H2A	120.2	C5—C4—H4	120.6
N1—C1—C2	123.08 (19)	N1—C5—C4	123.4 (2)
N1—C1—C6	117.54 (16)	N1—C5—H5	118.3
C2—C1—C6	119.38 (18)	C4—C5—H5	118.3
C1—C2—C3	118.2 (2)	O1—C6—N2	122.15 (18)
C1—C2—H2B	120.9	O1—C6—C1	122.66 (17)
C3—C2—H2B	120.9	N2—C6—C1	115.17 (17)
C4—C3—C2	119.2 (2)	H1WA—O1W—H1WB	102.7
			
C5—N1—C1—C2	0.3 (3)	C3—C4—C5—N1	−1.0 (4)
C5—N1—C1—C6	179.67 (17)	O2—N2—C6—O1	−0.4 (3)
N1—C1—C2—C3	−1.2 (3)	O2—N2—C6—C1	−178.54 (17)
C6—C1—C2—C3	179.4 (2)	N1—C1—C6—O1	175.6 (2)
C1—C2—C3—C4	1.0 (3)	C2—C1—C6—O1	−5.0 (3)
C2—C3—C4—C5	0.0 (4)	N1—C1—C6—N2	−6.2 (3)
C1—N1—C5—C4	0.8 (3)	C2—C1—C6—N2	173.20 (18)

### Hydrogen-bond geometry (Å, º)

**Table:**

*D*—H···*A*	*D*—H	H···*A*	*D*···*A*	*D*—H···*A*
O2—H2···O1*W*	0.82	1.86	2.656 (2)	163
N2—H2*A*···N1^i^	0.86	2.31	3.010 (2)	139
O1*W*—H1*WA*···O1^ii^	0.85	2.14	2.976 (2)	168
O1*W*—H1*WB*···O1^iii^	0.85	1.94	2.788 (2)	173

Symmetry codes: (i) −*x*+1, *y*, −*z*+1/2; (ii) *x*, *y*+1, *z*; (iii) −*x*+1, −*y*+1, −*z*+1.
